# Combinative Particle Size Reduction Technologies for the Production of Drug Nanocrystals

**DOI:** 10.1155/2014/265754

**Published:** 2014-01-06

**Authors:** Jaime Salazar, Rainer H. Müller, Jan P. Möschwitzer

**Affiliations:** ^1^Institute of Pharmacy, Department of Pharmaceutics, Biopharmaceutics and NutriCosmetics, Freie Universität Berlin, Kelchstraße 31, 12169 Berlin, Germany; ^2^Pharmaceutical Development, AbbVie Deutschland GmbH & Co. KG, Knollstraße, 67061 Ludwigshafen am Rhein, Germany

## Abstract

Nanosizing is a suitable method to enhance the dissolution rate and therefore the bioavailability of poorly soluble drugs. The success of the particle size reduction processes depends on critical factors such as the employed technology, equipment, and drug physicochemical properties. High pressure homogenization and wet bead milling are standard comminution techniques that have been already employed to successfully formulate poorly soluble drugs and bring them to market. However, these techniques have limitations in their particle size reduction performance, such as long production times and the necessity of employing a micronized drug as the starting material. This review article discusses the development of combinative methods, such as the NANOEDGE, H 96, H 69, H 42, and CT technologies. These processes were developed to improve the particle size reduction effectiveness of the standard techniques. These novel technologies can combine bottom-up and/or top-down techniques in a two-step process. The combinative processes lead in general to improved particle size reduction effectiveness. Faster production of drug nanocrystals and smaller final mean particle sizes are among the main advantages. The combinative particle size reduction technologies are very useful formulation tools, and they will continue acquiring importance for the production of drug nanocrystals.

## 1. Introduction

Standard techniques employed for the production of drug nanocrystals are high pressure homogenization and wet bead milling. These processes have been already employed to successfully formulate poorly soluble compounds [1]. However, they have drawbacks, such as long processing times and the necessity of employing a micronized drug as the starting material [2]. Next-generation technologies involve combinative particle size reduction methods to improve the particle size reduction effectiveness of the standard techniques [3]. There are basically two approaches to produce drug nanocrystals, with a third one (the combinative approach) being a combination of the first two techniques. The first process type produces drug nanocrystals by precipitating dissolved molecules. This approach is called bottom-up, as the size of the particles is increased. This group involves processes such as microprecipitation and chemical synthesis. The second process type involves particle size reduction or comminution. This approach is called top-down, as the size of already existing particles is decreased [4]. The third approach involves combinations of bottom-up and/or top-down steps to improve the particle size reduction effectiveness of the single-unit processes. The first step is usually a bottom-up process employed as a drug pretreatment to obtain a brittle, friable starting material for a subsequent comminution step. Thereby, the drug material is easier to nanosize. Combinations of two different top-down steps (such as bead milling followed by high pressure homogenization) have also been developed [5, 6].

## 2. Precipitation Processes (Bottom-Up)

The drug dissolved in a solvent is precipitated by adding an antisolvent. This is a classical precipitation process, also known as *via humida paratum *(Latin for “produced in a wet process”). The challenges of this technique are to minimize the crystal growth into the nanometer range (controlled crystallization) and to control the solid state of the crystals, that is, to produce them in crystalline or amorphous form [5].

The “hydrosols” technology is the first process involving a bottom-up step to produce drug nanoparticles; this technology was developed by Sucker and nowadays belongs to Novartis [7, 8]. The process has the advantage of producing crystalline drug nanoparticles. However, this technology has a set of drawbacks. The drug has to be soluble in at least one solvent and the process involves organic solvents that need to be removed. There are apparently no products on the market that use this technology, perhaps due to the difficulty of avoiding the crystal growth.

Another precipitation technology is the Nanomorph process developed by Auweter et al. [9, 10]. This technology yields amorphous drug nanoparticles, which have the advantage of higher saturation solubility and a faster dissolution rate compared to the crystalline form. However, drawbacks include undesired compound recrystallization to the crystalline state with a subsequent decrease in bioavailability [11].

Another bottom-up technology is the controlled crystallization during freeze-drying [12]. This technology involves a precipitation process (lyophilization) to produce nanocrystalline particles. Subsequently, the freeze-dried powders can be directly employed to manufacture tablets. This makes it a simple single-unit precipitation process. Additionally, drug releases of up to 80% after 10 min of dissolution testing were reported. The special features of this bottom-up technology are the production of crystalline drug nanoparticles and the ability for large-scale production [12, 13].

Freeze-drying as well as spray-drying are precipitation processes widely employed in the pharmaceutical industry to obtain dry intermediates or final drug powders. Both bottom-up technologies can also be employed to modify drug materials to make them more suitable (i.e., friable and brittle) for a subsequent comminution process [3].

## 3. Comminution Processes (Top-Down)

### 3.1. Wet Bead Milling (WBM, NanoCrystal Technology)

The pearl or bead milling technique was developed by Liversidge et al. [14] and is owned by Alkermes plc. It is referred to as the first-generation production method for drug nanocrystals. This technology comprises a milling chamber with an agitator, which is loaded with the milling material, a dispersium medium (usually water), surfactants as a stabilization system, and the drug to be nanosized. The drug particles are reduced in size by mechanical attrition. The milling material is usually small beads of stainless steel, glass, ceramic (e.g., yttrium stabilized zirconium dioxide), or highly cross-linked polystyrene resin, the last two being preferred due to reduced contamination to the product. The milling pearls have different sizes (e.g., 0.1, 0.2, or 0.5 mm). The collision frequency during the comminution process is increased with the reduction in size of the milling beads. Thus, the particle size reduction effectiveness can be enhanced [15]. Other factors affecting the comminution effectiveness are the hardness of the drug, the surfactant and its concentration, temperature, the viscosity of the dispersion medium, and so forth. The forces producing the particle size reduction include shear forces, and particle collision produced by the movement of the milling material inside the chamber. The NanoCrystal technology is regarded as a successful technology: the first product containing drug nanocrystals (Rapamune by Wyeth Pharmaceuticals in 2000) came to the market only 10 years after the development of the technology [16, 17].

### 3.2. High Pressure Homogenization (HPH)

The HPH technique is a high-energy disintegration process that employs high pressure to reduce the particle size of drug particles in liquid media with surfactants for stabilization purposes. The HPH involves the principles of piston-gap homogenization and jet-stream homogenization (microfluidization) [1, 18].

#### 3.2.1. Piston-Gap Homogenization

When the piston-gap HPH technique is employed, the particle size reduction is achieved by cavitation, shear forces and particle collision. The suspension is forced through a small gap, which reduces the diameter from 3 cm to approximately 25 *μ*m [19]. Because of the tremendous diameter change, according to Bernoulli's law, the dynamic pressure raises and the static pressure falls. As a result of the latter, the liquid starts boiling in the homogenization gap, as the static pressure is lower than the vapor pressure of the liquid. This phenomenon results in the formation of gas bubbles that implode after leaving the gap (cavitation). The shear forces and the particle collisions are developed during the process due to the high pressures involved (usually up to 1500 bar). The equipment employed for piston-gap homogenization is produced, for example, by APV, Gaulin and Avestin [18, 20].

Employing piston-gap homogenizers, Müller and coworkers developed the Dissocubes technology (now belonging to Skyepharma plc) and the Nanopure technology (now belonging to Abbott GmbH & Co. KG) [18, 21]. These technologies produce drug nanocrystals by employing high pressures of up to 1500 bar. However, they employ different process media for the homogenization process. The Dissocubes technology produces drug nanoparticles in an aqueous dispersion at room temperature. On the contrary, the Nanopure process employs nonaqueous media (e.g., oils or liquid polyethylene glycols) or water-reduced media (e.g., employing glycerol/water mixtures) [2]. One interesting feature of the Nanopure technology is that the oil dispersions can be employed to subsequently fill capsules as the final dosage form [5].

#### 3.2.2. Jet-Stream Homogenization (Microfluidization)

The microfluidization technology (Microfluidizer, Microfluidics Inc., USA) is based on the jet-stream principle. The drug is suspended in aqueous media with surfactants for stabilization. Then, the suspension is put into the homogenizer device for processing. During the homogenization process, two jet streams are forced to circulate at high pressure (up to 1700 bar) through two different interaction chambers (*Y* and *Z*). This produces particle collision and shear forces as well as cavitation, which results in the disintegration of the drug particles [22]. SkyePharma Canada Inc. employs a Microfluidizer homogenizer for its IDD-P (insoluble drug delivery particles) technology, which produces submicronic nanosuspensions [23].

## 4. Combinative Technologies

In summary, the known limitations of the standard processes (WBM, HPH) for the production of drug nanocrystals are the necessity of a micronized drug as the starting material and the long runtimes for the top-down equipment [2]. The combinative particle size reduction techniques have been developed to overcome these drawbacks and to improve the particle size reduction effectiveness of the standard processes. Nowadays, five combinative methods are known: NANOEDGE (microprecipitation followed by a high-energy step such as HPH), H 69 (microprecipitation immediately followed by HPH, also called “cavi-precipitation”), H 42 (spray-drying followed by HPH), H 96 (freeze-drying followed by HPH), and the CT combinative technology (media milling followed by HPH) [5]. These technologies are described later in this paper. The applications of the combinative technologies for a variety of drugs are shown in Table 1.

### 4.1. NANOEDGE Technology

The NANOEDGE technology from Baxter is the first combinative particle size reduction method developed for the production of drug nanosuspensions. This production technique combines a microprecipitation step (a solvent-antisolvent technique) followed by a high-energy process. The drug is first dissolved in a suitable solvent, usually a water-miscible organic solvent. The drug solution is then mixed with a second aqueous liquid in which the drug is less soluble. The aqueous liquid can contain surfactants for stabilization, and it is added to the drug solution in a controlled manner using, for example, an infuser device. Subsequently, the precipitation occurs due to the change in solubility. The microprecipitation is a pretreatment and the drug particles can be obtained in amorphous or semicrystalline form. Then, the drug particles are reduced in size and transformed to the more stable crystalline state after employing a high-energy annealing step, such as high pressure homogenization [24, 25]. The objective of the annealing step is to improve the thermodynamic stability of the nanosuspensions by preventing the crystal growth of the precipitated particles to the micrometer range. This is achieved by single or repeated energy applications, followed by thermal relaxation. The change to the more stable form is produced by the high-energy input of the top-down step, which promotes the formation of low-energy, more stable structures, either by enhancing the crystallinity of the particles (reordering of the lattice structure) or by rearrangement of the stabilizing system at the surface of the drug nanocrystals. The fast microprecipitation improves the particle size reduction effectiveness of the top-down step due to induced friable material, drug crystal defects, and dendritic morphology. The top-down process is usually high pressure homogenization but other techniques such as sonication or microfluidization can also be employed [25, 26].

The residues of organic solvents in the nanosuspension are a major problem associated with this combinative technology, which becomes more complicated in the case of large-scale production (i.e., larger amounts of solvent to be removed from the final drug product). This is difficult when a nanosuspension is needed. Another drawback is that this technology achieves particle sizes markedly bigger than with standard technologies. As the Baxter development is mainly focused on injectables, the solvent-removal processes as well as the production lines need to be performed under sterilized conditions. This situation makes the process more complicated and expensive. This technology has no marketed products to date, as I.V.-injectable products are more complicated to develop as oral products [5].

#### 4.1.1. NANOEDGE Applications

The NANOEDGE platform has been employed to formulate poorly soluble anticancer drugs such as paclitaxel to improve the plasma concentration and thereby the pharmacologic efficacy. Nanosuspensions formulated with 1–5% (w/v) drug presented particle sizes of around 1000 nm. The plasma levels achieved by the antineoplastic drug nanocrystals were tested in animal models. The drug nanosuspensions produced by this technology could achieve higher drug loads and a more flexible administration, such as oral and injectable routes [27].

The therapy efficacy for an anticancer compound described as “A” was established in rat tumor models. The nanosuspension formulation showed better tolerability in rats than the drug formulated with standard techniques, which enable the implementation of higher doses. However, the efficacy of the tumor suppression was comparable after I.V. injection and after oral administration. With another poorly soluble, poorly bioavailable anticancer drug described as “B,” a linear relationship was found between the decreasing in nanosuspension particle size (particle sizes of 400–1000 nm, administered at 300 mg/kg) and the increase of oral bioavailability measured from the plasma of rats. In this case, the formulation as a nanosuspension employing the NANOEDGE process resulted in an up to 30-fold bioavailability increase in the rat models compared to the control formulation [27].

The NANOEDGE combinative technology was also employed to reformulate a paclitaxel product (Taxol, Bristol Myers Squibb Company). The objective was to eliminate Chremophor EL (polyethoxylated castor oil) as an excipient to avoid its incompatibilities and toxicity. Functionalized polyethylene glycols were employed as surfactants to minimize the opsonization of the drug nanocrystals, which had a mean particle size of 200 nm [27, 28].

The poorly soluble drug itraconazol was processed employing the NANOEDGE technology. It was dissolved in N-methyl-2-pyrrolidone (NMP) and then precipitated by adding an aqueous diluent with surfactants. Sonication for one minute at 10000 Hertz (Hz) and 400 watts (W) employed as the annealing step resulted in drug nanoparticles with a mean particle size of 177 nm [25].

In another study, an itraconazol nanosuspension for I.V. administration was developed employing the NANOEDGE process. Its resulting bioavailability was compared to the results of a marketed itraconazol solution (Sporanox IV, Janssen Pharmaceutica), which is formulated with cyclodextrin technology and presents some degree of toxicity due to the high cyclodextrin load. In this case, HPH was used as the annealing step, achieving a final mean particle size of 581 nm. Subsequently, in vivo studies were performed in rat models. The nanosuspension formulation led to better bioavailability and tolerability, enabling the use of higher drug doses. The subject survival was superior with the nanoparticulated itraconazol due to higher drug concentrations in the target organs compared to the standard solution formulation [28, 29].

Carbamazepine, prednisolone, and nabumetone were also processed with this technology. The drugs were separately dissolved in NMP and then precipitated by adding distilled water. Carbamazepin and prednisolone presented a needle-shaped form and a mean particle size of approximately 2 *μ*m after precipitation. An Avestin C50 homogenizer (Avestin Inc., Canada) was employed for the high-energy step to process the three drug macrosuspensions, which resulted in final mean particle sizes of 400 nm for carbamazepin, 640 nm for prednisolone and 930 nm for nabumetone [25].

There are also a variety of drugs processed by “Nanoedge-like” processes involving a microprecipitation step followed by a high-energy process (HPH or sonication), although they are not addressed as “Nanoedge.”

The antitumor alkaloid 10-hydroxycamptothecin (10-HCPT) was processed by employing a microprecipitation-homogenization process. The drug was dissolved in dimethyl sulfoxide (DMSO), and then it was precipitated by adding an aqueous surfactant solution. The drug suspension was then homogenized employing an ATS AH110D piston-gap homogenizer (ATS Engineer Inc., China). The drug particles were obtained in the amorphous state and the best final mean particle size of 131 nm was obtained by homogenizing the drug suspension for 20 cycles at 1000 bar [30].

Isradipine was also processed employing a microprecipitation-HPH technique. The drug was dissolved in 2-propanol and then precipitated by adding an aqueous solution containing surfactants for stabilization purposes. This macrosuspension was then processed by HPH employing a piston-gap homogenizer (GEA Niro Soavi Inc., USA) for 30 cycles at 1200 bar. The resulting nanosuspension had a mean particle size of 469 nm [31].

The nonsteroidal anti-inflammatory drug meloxicam was also processed employing a combinative approach. This drug was dissolved in dimethylformamide (DMF) and then precipitated by adding the drug solution to an aqueous solution containing surfactants. The drug particles were then further processed either by ultrasonication (20 min 300 W with a FS-5 sonicator, Frontline Ltd., India) or by HPH (15 cycles at 500 bar with an ATS AH110D homogenizer). The results showed a final mean particle size of 259 nm with the sonication method and 212 nm with the HPH technique. However, the amount of larger crystals was considerably smaller when HPH was used as the reduction step. Additionally, low pressure could be maintained (500 bar), as higher pressures (or a higher number of homogenization cycles) did not improve the particle size reduction [32].

In the case of nitrendipine, the drug was processed by employing a microprecipitation-ultrasonication process. The drug was first dissolved in a 1 : 1 PEG 200 : acetone mixture, and then it was precipitated by adding a polyvinyl alcohol aqueous solution. The drug particles were subsequently processed by employing ultrasonication (Ningbo Scientz Biotechnology Co. Ltd., China). The best mean particle size result of 209 nm was obtained by employing 20000 Hz and 400 W as sonication conditions for 15 min. Nitrendipine suffered no substantial crystallinity change after the process [33].

All-trans retinoic acid is a poorly soluble, heat-sensitive, anticancer drug. A microprecipitation-sonication process under controlled temperature was chosen to produce nanoparticles of the drug to eventually improve its dissolution rate-dependent bioavailability. The drug was first dissolved in acetone, and then it was mixed with demineralized water to produce the precipitation. The drug particles were immediately sonicated employing an EQ-250E medical ultrasonicator (Kunshan Ultrasonic Instrument Corporation, China) for 30 min. A final mean particle size of 155 nm was obtained. However, this result was only slightly improved compared to the precipitation process without sonication (176 nm mean particle size). Additionally, the precipitation process produced, in general, amorphous drug particles [34].

Hydrocortisone was processed performing experiments with a microprecipitation-sonication technique. This drug was dissolved in ethanol and then precipitated by adding an aqueous surfactant solution. The drug particles were immediately sonicated after precipitation for 5 min. The process factors that were investigated included solvent:antisolvent flow rate and drug concentration. A mean particle size of 80 nm could be achieved under optimized conditions. The precipitation process modified the high crystallinity of the starting material leading to amorphous drug nanoparticles [35, 36].

Finally, a microprecipitation-sonication process was performed with ibuprofen. The drug was dissolved in acetone and then added to an aqueous solution containing surfactants. The precipitated drug particles were further sonicated for 60 min employing a Sonic Dismembrator model 550 (Fisher Scientific International Inc., USA). Different surfactants were screened for the precipitation step and the best stabilization results were achieved with HPMC K3, which led to a mean particle size of 702 nm after sonication [4].

### 4.2. H 69 Technology

The H 69 process was developed by Müller and Möschwitzer, and it belongs to the smartCrystal technology family. This combinative process is similar to the NANOEDGE approach. It combines a microprecipitation step involving organic solvents, followed by high pressure homogenization for particle size reduction. The difference is that with the H 69 technology, the cavitation takes place at the same time as the particle formation (“cavi-precipitation”) or at most two seconds thereafter. To employ this combinative technique, the drug is dissolved in a suitable solvent (liquid 1), which is then mixed with an aqueous nonsolvent (liquid 2). The nonsolvent is added to the solvent in a controlled manner using, for example, an infuser device such as the Perfusor from B. Braun Melsungen, Germany. To do this, different pump rates can be adjusted. The liquid flows come in contact, which results in the precipitation of the drug. The particle formation takes place in the high-energy zone of a homogenizer, where the just-formed drug particles are immediately treated with cavitation, particle collision, and shear forces. The Microfluidizer or the EmulsiFlex C5 from Avestin are suitable homogenizers to process the liquid flows directly in the high-energy zone of the device [37].

As with all precipitation methods, the challenge is to control the particle crystallization by avoiding crystal growth. Nucleation can be stopped by employing this “cavi-precipitation” technique, where the drug particles formed are immediately treated with a high-energy annealing process. The top-down step not only reduces the particle size but also stabilizes the drug nanocrystals with the energy application. Another advantage of the annealing step is that it promotes the more stable crystalline form [37]. See Section 4.1 for more information about the annealing step. A drawback of this combinative process is that the resulting nanosuspensions contain organic solvent residues that need to be removed before further processing, just as with the NANOEDGE technology.

#### 4.2.1. H 69 Applications

Prednisolone was processed employing this combinative technology. The drug was dissolved in ethanol, mixed with demineralized water as a nonsolvent for precipitation, and then directly homogenized at high pressure. A mean particle size of 113 nm could be achieved after one minute of homogenization. These results improved to 27 nm after 5 min and to 22 nm after 6 min. Afterwards, the drug nanocrystals dissolved due to the increased dissolution pressure at these small particle sizes [37].

The drugs hydrocortisone acetate (HCA) and omeprazol were processed employing the H 69 process, achieving mean particle sizes of 787 nm and 921 nm, respectively, after 20 cycles of homogenization at 1500 bar [37].

Ibuprofen and resveratrol are other examples of drugs processed with the H 69 technology. In the case of ibuprofen, best results were achieved when the drug was dissolved in tetrahydrofuran and then precipitated by adding demineralized water with surfactants. These drug particles then showed a mean particle size of about 10 *μ*m. The drug crystals were immediately homogenized employing a Micron LAB 40 device (APV Gaulin, Germany) for 10 cycles at 1500 bar or the EmulsiFlex C5 homogenizer for 10 cycles at 1200 bar. The latter equipment produced the smallest ibuprofen nanocrystals, which presented a mean particle size of 170 nm. The EmulsiFlex C5 has the advantage that the precipitated drug particles can be directly homogenized at the high-energy zone of the device. Thus, it is possible to immediately stabilize the drug nanocrystals to ensure small particle sizes. In the case of resveratrol, best results were achieved by dissolving the drug in a DMSO/acetone mixture. The drug was then processed with the Avestin C5 as described for ibuprofen. A final mean particle size of 150 nm could be achieved in the case of resveratrol. The reduction of the time between precipitation and the top-down step and the proper selection of the organic solvent to dissolve the drug prior to the precipitation step were identified as critical factors in obtaining the smallest drug nanocrystals [38].

### 4.3. H 42 Technology

The H 42 process was developed by Möschwitzer, and it also belongs to the smartCrystal technology platform. This combinative technology combines spray-drying (SD) as a precipitation and pretreatment step, followed by HPH for particle size reduction. The organic solvent is eliminated during the bottom-up step, which differentiates this technology from the NANOEDGE and H 69 processes.In the first unit operation (SD), the poorly soluble compound is dissolved in organic solvents. Surfactants such as poloxamer or sugars such as mannitol can be added to the drug solution to improve the results of the drying step. The solvent selection is critical to improve the performance of the process. The ideal organic solvent should possess good dissolving properties as well as suitable both boiling point and vapor pressure to ensure an efficient process and spray-dried powders free of solvent residues. Additionally, the selected solvent should ideally possess a low toxicity [3].

The objective of the drug modification by means of SD is to produce suitable, more breakable drug powders for the subsequent comminution process. The obtained spray-dried drug powders are then dispersed in aqueous media containing surfactants for stabilization purposes. The suspensions are further processed to nanosuspensions by employing the HPH technique, using homogenization equipment such as the Micron LAB 40 [39].

The H 42 combinative technology has advantages such as relatively short processing times during SD, solvent-free dry intermediates, and small drug nanocrystals after a reduced number of HPH cycles. Its drawback is the employment of high temperatures during SD, which could make this technology unsuitable to process thermolabile compounds.

#### 4.3.1. H 42 Applications

In the first experiments, ibuprofen was processed employing this combinative technology. The drug was dissolved in ethanol and then spray-dried. The modified powders were then homogenized for 20 cycles at 1500 bar, reaching a mean particle size of 636 nm (original value without modification: 1172 nm). The spray-dried ibuprofen powders showed almost no crystallinity change compared to the unprocessed material, which was confirmed by employing the differential scanning calorimetry (DSC) technique. The melting points and the normalized melting enthalpies of unmodified and spray-dried modified ibuprofen were compared and showed almost no difference. In this case, the improved reduction effectiveness was not linked to a change in the solid state behavior of the drug, but to the enhanced friability of the starting material [39].

Amphotericin B was also processed employing the H 42 technology. This model compound was dissolved in a 1 : 19 DMSO/methanol mixture and then spray-dried. The drug powders were homogenized at 1500 bar for 20 cycles using PEG 300 as a dispersion medium with the purpose of employing the nanosuspension to directly fill capsules. The process yielded a final mean particle size of 172 nm [39].

The model compound HCA was also processed employing the H 42 technology. The drug was dissolved in ethanol, with different amounts of poloxamer 188 being added to the drug solution. The spray-dried drug processed with a 9 : 1 drug/surfactant ratio brought the best particle size results, which were 281 nm after 20 homogenization cycles at 1500 bar. Additionally, this finely dispersed nanosuspension presented high storage stability. The micronized, unmodified HCA led to a final mean particle size of 551 nm under the same process conditions [2].

The improved drug structure of the best spray-dried powder was analyzed employing the scanning electron microscopy (SEM) technique, which showed spherical drug particles. Further, the solid state behavior of the spray-dried powders was analyzed by using the powder X-ray diffraction (PXRD) technique. These results showed that the spray-dried powders stayed as crystalline as the unmodified drug material. The SD process did not modify the crystallinity of HCA. Small amounts of the surfactant positively impacted the characteristics of the spray-dried powders, such as flowability and millability. On the contrary, high surfactant amounts (i.e., 1 : 1 drug/surfactant ratio) negatively impacted the powders' characteristics and the subsequent particle size reduction effectiveness. The processing times could also be drastically reduced. When the best modified material was employed, only one cycle at 1500 bar was necessary to achieve smaller particle sizes than by homogenizing micronized drug material for 20 cycles [2].

Glibenclamide was further processed employing the H 42 technology. The influence of both surfactant and drug concentration during the bottom-up step was tested in this study. The effect of these parameters on the solid state behavior and morphology of the drug, as well as on the particle size reduction effectiveness of the top-down step, was analyzed. The degree of crystallinity (DC) of the drug powders was established employing the DSC technique. It was discovered that the spray-dried glibenclamide powders showed, in general, a reduced crystallinity (DCs of between 20% and 30%) compared to the unmodified drug (100% DC). However, the drug solutions processed with medium and high drug concentrations (both sprayed with a 0.2% docusate sodium salt ethanolic solution) produced spray-dried powders with very low DCs: 8.1% and 8.3%. Both powders led after the homogenization step to nanosuspensions presenting mean particle sizes of about 236 nm, which were the best of all the results. Additionally, the SEM analysis of these glibenclamide samples revealed the formation of spherical drug particles. Both solid state modification leading to an amorphous drug and the morphology change due to the precipitation process positively impacted the particle size reduction effectiveness of the top down-step [3].

With the antioxidant compound resveratrol, experiments were also performed with the H 42 process employing a design of experiments. Resveratrol was dissolved in ethanol containing different amounts of the surfactant sodium cholate, and then the drug solutions were spray-dried for further homogenization. The best mean particle size obtained was 200 nm, which was an improvement compared to the 428 nm mean particle size obtained with unmodified resveratrol. Additionally, the amount of larger crystals was drastically reduced by employing the spray-dried modified drug instead of micronized material: from 2.2 *μ*m (d90%) to 0.736 *μ*m (d90%). Finally, the number of HPH cycles at 1500 bar necessary to achieve a proper nanosuspension could be reduced from 20 cycles with the standard method to only one cycle with the modified drug. This is one of the most important features of the H 42 technology. However, it was difficult to establish a link between the DC and the smallest drug particle sizes with resveratrol as the model drug [40].

### 4.4. H 96 Technology

The H 96 combinative technology was developed by Möschwitzer and Lemke and belongs to the smartCrystal technology family (Abbott/Soliqs, Germany). This process involves freeze-drying (FD) as a bottom-up and pretreatment step, followed by HPH for particle size reduction. The bottom-up step eliminates the organic solvent content, just as with the H 42 technology. The FD step involves the dissolution of poorly soluble drugs employing organic solvents. The drug solution is then frozen (e.g., with instant freezing or snap-freezing) with liquid nitrogen and further freeze-dried. The aim of the drug pretreatment is to modify the starting material to improve the particle size reduction effectiveness of the HPH [5, 41].

The solvents need to be carefully selected to optimize the process and the characteristics of the freeze-dried powders. The critical solvent characteristics that determine the process performance are, among others, the freezing point, vapor pressure, and toxicity. For FD purposes, it is important to employ organic solvents presenting relatively high freezing points. In this way, it is ensured that the solvent crystallizes completely during the lyophilization process. The selected solvent should also possess a high vapor pressure to ensure a complete elimination during the primary drying step. The complete removal of residues of organic solvents is necessary to ensure patient safety and product quality [42, 43].

Mixtures of organic solvents can also be implemented to improve the performance of the lyophilization process. For example, experiments using glibenclamide as a model compound had employed mixtures of dimethyl sulfoxide (DMSO) and tert-butyl alcohol (TBA) for FD. DMSO dissolves the model compound but has a low vapor pressure, which resulted in low-quality freeze-dried powders (i.e., wet and sticky due to incomplete elimination of the solvent). TBA has both a high freezing point and vapor pressure, which makes it an ideal solvent for lyophilization purposes. DMSO contributed to the process with the necessary dissolving force for glibenclamide, and TBA was added to the solution to improve the characteristics of the freeze-dried cakes [43, 44].

The H 96 technology is especially suitable to process thermolabile or expensive drugs due to the low temperatures and the high yields of the FD. Additionally, as the lyophilization step eliminates the organic solvent content, the subsequently produced nanosuspensions are ready to be further processed or used. Its drawback is the extension of the lyophilization step.

#### 4.4.1. H 96 Applications

During first experiments employing the H 96 technology, amphotericin B was dissolved in DMSO, snap-frozen with liquid nitrogen, and then lyophilized. The freeze-dried drug powder was processed to a nanosuspension employing a Micron LAB 40 homogenizer for five cycles at 1500 bar producing drug nanocrystals of a 62 nm mean particle size [41]. The snap-freezing or instant freezing with liquid nitrogen was necessary to achieve this very low particle size, as slowly freezing the drug solution resulted in bigger particle sizes after the top-down step (186 nm). In addition to the ultrasmall particle size, the process became extremely cost-effective by reducing the number of homogenization cycles from 20 with the standard technique to only one cycle at 1500 bar with the combinative technology. In another study, human erythrocytes were loaded with an amphotericin B nanosuspension produced with the H 96 technology. The antifungal treatment could be improved due to the enhanced pharmacological profile of the amphotericin B nanocrystals [45, 46].

HCA was also processed as described for amphotericin B. After dissolving the drug in DMSO, the drug solution was snap-frozen with liquid nitrogen. The drug powder was processed to a nanosuspension employing the HPH technique for 10 cycles at 1500 bar. The final mean particle size was 414 nm for this drug nanosuspension. Another drug processed with the H 96 process was cyclosporine A. This compound was dissolved in a 1 : 1 ethanol : DMSO mixture, freeze-dried employing the snap-freezing technique, and further homogenized for 15 cycles at 1500 bar. A mean particle size of 440 nm was reported in this case [41].

Further experiments employing glibenclamide revealed a relationship between the crystallization conditions and the particle size reduction effectiveness of the top-down step. The different ratios of a DMSO-TBA mixture (90 : 10 to 10 : 90 v/v) and the drug concentration during the bottom-up process modified the solid state behavior of the drug as well as its morphology. The micronized and freeze-dried glibenclamide powders were analyzed with the DSC technique to determine their DC. The micronized glibenclamide possesses a DC of 100% while most of the lyophilized powders showed DCs between 50% and 60%. However, when a design of experiments for the assessment of the critical crystallization factors was employed, it was found that solvent mixtures containing a high TBA proportion (i.e., DMSO : TBA : 10 : 90 v/v) and a low drug concentration favored the formation of highly amorphous glibenclamide. This modified drug powder showed a DC of 1%. It was found that the H 96 technology is able to produce drug powders in either a crystalline or an amorphous state, depending on the process conditions and additives. Additionally, the process conditions modified the morphology of glibenclamide from a hard, rough structure to a fine, subtle, and brittle structure, determined by the SEM technique. The drug modification by means of FD was advantageous for the HPH step. A mean particle size of 164 nm could be obtained under optimized conditions, which was markedly improved compared to the unmodified glibenclamide (772 nm). The homogenization length could also be reduced from 20 cycles to only one cycle, which was sufficient to produce a nanosuspension with a smaller particle size than after 20 cycles with the standard method [44].

In another study also employing glibenclamide as a model drug, the comminution effectiveness of the WBM and HPH processes when employing lyophilized drug as a starting material was compared. The FD solvents were DMSO : TBA mixtures prepared with solvent ratios of 90 : 10 to 10 : 90 (v/v). The drug concentration was kept constant at 5% for both bottom-up and top-down steps. Both methods were an improvement over the standard process using unmodified material. In the case of the WBM, the process time was reduced from 24 hours to only one hour to achieve a proper nanosuspension. Smaller particle sizes can be achieved much faster by modifying the drug structure. In the case of the HPH, the number of homogenization cycles was reduced from 20 to only five cycles to achieve a sufficiently small particle size. A mean particle size of 160 nm was reported employing WBM on modified material after 24 hours of processing. In addition, a mean particle size of 335 nm was obtained using HPH on a freeze-dried modified drug. Both methods benefited from the freeze-dried drug modification. However, the processes benefited from different drug characteristics. For the WBM process, the higher friability and volume of the drug powders, which remained crystalline, were beneficial. For the homogenization process, the change in the drug crystal behavior from crystalline to amorphous to achieve smaller particle sizes was beneficial. This feature was confirmed by assessing the DC of the drug powders employing the DSC technique [42].

### 4.5. Combination Technology (CT)

The CT technology is the only combinative process that does not employ organic solvents. The CT process combines a low-energy pearl milling step, followed by high pressure homogenization for particle size reduction. The shear forces and particle collision are combined with the cavitation for an innovative particle size reduction process [6]. The pretreatment of the drug involves the milling of its macrosuspension. This step achieves, in general, drug particle sizes between 600 nm and 1500 nm. The subsequent homogenization process improves the homogeneity of the nanosuspension by reducing the particle size and the amount of larger crystals. The latter feature also enhances the physical stability by avoiding crystal growth (Ostwald ripening), which improves the long-term stability of the drug nanosuspensions during storage [47]. Interestingly, it was reported that lower homogenization pressures (100–500 bar) resulted in smaller drug nanocrystals and more homogeneous nanosuspensions than higher homogenization pressures (1500 bar) after the pearl milling step [6]. The advantages of this technology are the reduction of the homogenization pressure and process length, as well as the improved physical stability of the nanosuspensions. However, the CT process leads to particle sizes that are relatively bigger compared to the other combinative technologies.

#### 4.5.1. CT Applications

The flavonoid hesperidin is an example of a poorly soluble drug processed with the CT technology. A mean particle size of 599 nm was reported for hesperidin nanosuspensions, which also showed improved long-term stability [48]. Special features of the production of nanoparticulated hesperidin employing the CT process were the reduction of the homogenization cycles (from 20 to five) and of the necessary pressure (from 1500 bar to 1000 bar) to achieve a nanosuspension. In this manner, it is possible to reduce the energy input and the wearing of the machines [6].

Rutin and apigenin are also poorly soluble drugs processed with the CT technology. These drugs are flavonoids showing antioxidant properties with potential applications in pharmaceutical and cosmetic products. The first cosmetic product formulated employing nanotechnology contains rutin nanocrystals and was launched by Juvena, Switzerland [47]. Hesperidin nanocrystals can be found in the Platinum Rare cosmetic product (La Prairie, Switzerland) [18]. The topical route has been reported as full of potential for nanoparticulate applications, as the drug nanocrystals enhance the compound penetration to the skin. Furthermore, the CT technology produces drug nanosuspensions with increased stability again electrolytes. The electrolytes could lead to aggregation by reducing the zeta potential (i.e., the electrostatic repulsion) of the drug nanocrystals, thus producing the loss of their fast dissolution properties [6].

Employing apigenin, the CT technology led to a final mean particle size of 275 nm after only one homogenization cycle at 300 bar using an Avestin C50 homogenizer. The pearl-milled product presented a mean particle size of 412 nm, which was further reduced by the homogenization step. Interestingly, in this case lower pressures had an advantage in achieving smaller drug nanocrystals.

In the case of rutin, a suspension of the drug was pearl-milled with zirconium oxide beads (0.3 mm) to a mean particle size of about 1000 nm. This premilled suspension was then homogenized employing the Avestin C50 for one cycle at different pressures. The best mean particle size of 604 nm was achieved employing low pressure (100 bar).

An up-scaling with apigenin was also performed using this technology. The nanosuspension production could be scaled from a 20 g batch to a 3 kg batch. The milling process was performed using an agitating pearl mill Bühler PML 2 (Bühler AG, Switzerland) with zirconium oxide beads (bead size: 0.4–0.6 mm). The homogenization part of the CT process was performed employing the Avestin C50 for one cycle at 300 bar. The premilling step resulted in drug particles with a mean particle size of 413 nm, which remained constant after the homogenization process. However, the homogenization produced a narrowing of the particle size distribution, manifested through a decreasing polydispersity index. This feature is critical to enhance the physical stability of the nanosuspensions. The particle size, crystallinity, and physical stability of the nanosuspension were maintained when up-scaling the process, which is necessary for industrial production. Further, the CT technology could drastically reduce the number of homogenization cycles to just one, which is more cost-effective [49].

## 5. Technologies Evaluation: Comparison between Combinative Processes

**Figure 1:**
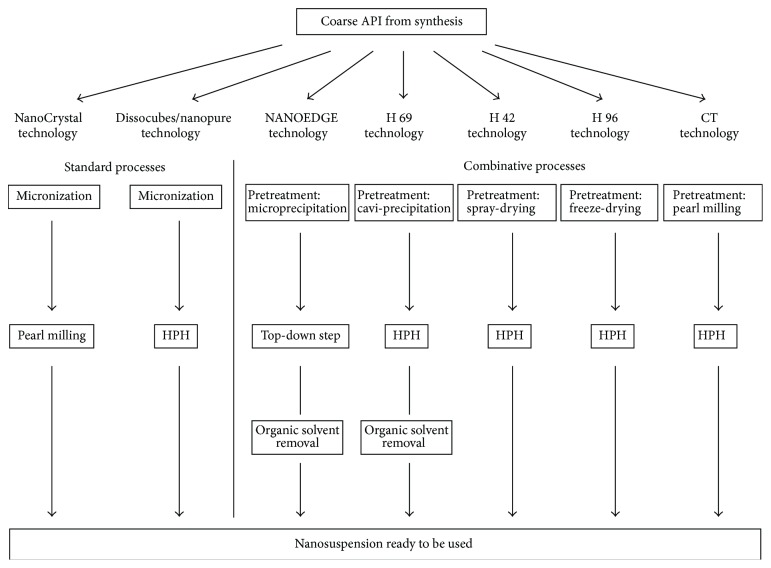
Schematic description of standard and combinative particle size reduction technologies.

New formulation technologies are the key to overcoming the increasing problem of poor aqueous solubility among emerging compounds [50]. The combinative particle size reduction processes have been presented as a part of the new enabling technologies. A schematic description of the standard particle size reduction processes (left side) and the combinative technologies (right side) is shown in Figure 1. Employing the combinative methods, the micronization step is replaced by a pretreatment [2].

Combinative processes such as the H 42, H 96, H 69, and the NANOEDGE technologies enable the direct processing of a drug solution after synthesis without previously performing a crystallization step. However, as the H 69 and NANOEDGE technologies involve the precipitation of particles in liquid media that usually contain organic solvents, these nanosuspensions are not ready to be used further (see Figure 1). Extra drying steps need to be performed to eliminate the organic solvent content, which makes the process longer, more expensive, and more complicated regarding regulatory aspects [5, 43]. On the contrary, when employing the H 42 and H 96 technologies, the organic solvent necessary to dissolve the poorly soluble drugs is eliminated during the bottom-up step. In this manner, the nanosuspensions produced with the dried intermediates can be directly used or down-streamed for the production of solid dosage forms [5, 18, 51].

A wide variety of drugs processed with the combinative technologies is shown in Table 1. These results were also described in the respective chapter of the combinative technologies. The NANOEDGE technology is the only process with a main focus on injectables (I.V. administration). The other technologies are focused on nanosizing for dissolution rate improvement for oral administration or formulation for topical administration. The formulation for cosmetic and nutraceutical applications, such as those discussed by Petersen in the CT technology patent, has also been successful [6].

When comparing the production length of the processes, the NANOEDGE and the H 69 techniques are relatively fast due to the rapid precipitation step. However, the organic solvent content of the nanosuspensions needs to be removed when employing these technologies, and so they lose the advantage of producing nanosuspensions in a fast process. The H 96 technology is more time-consuming due to the lengthy FD process. However, the lyophilization technique results in yields near to 100%, which is important in the case of expensive compounds. Additionally, the H 96 nanosuspensions do not contain amounts of organic solvents, which enable their direct usage after production. Finally, the H 42 technology produces nanosuspensions in a fast process. The SD is a rapid production step that can be performed in continuous mode. The H 42 nanosuspensions can also be subsequently directly processed or used, as they do not contain organic solvents.

Regarding the particle size reduction effectiveness, the H 96, H 69, and H 42 technologies are the processes achieving the smallest particle sizes for a variety of drugs (Table 1). Also, some microprecipitation-high-energy approaches (NANOEDGE and “Nanoedge-like”) led to small mean particle sizes.

By processing the same drug with different combinative techniques, it is possible to compare the reduction effectiveness and performance of the technologies. For example, amphotericin B and glibenclamide were both more effectively processed with the H 96 technology than with the H 42 process. In the case of amphotericin B, final mean particle sizes of 62 nm and 172 nm were achieved employing the H 96 and H 42 technologies, respectively [39, 41].

In the case of glibenclamide, final mean particle sizes of 164 nm and 236 nm were achieved employing the H 96 and H 42 technologies, respectively. The factors influencing the particle size reduction effectiveness were the porosity and the crystallinity of the drug powders. Both technologies produced drug powders with porous and brittle drug structures as well as with modified crystallinity. The H 96 technology produced, under optimized conditions, glibenclamide powders with 1% DC, which subsequently led to the low mean particle size of 164 nm. In comparison, the H 42 process led to glibenclamide powders with relatively higher DC (8.1%), which also resulted in relatively bigger particle sizes after the homogenization step (236 nm). However, both technologies achieved homogeneous dispersed nanosuspensions with a low particle size. Additionally, the H 42 technology had the advantage of being a much faster process [3, 44].

When comparing the H 42 and H 69 performances, the results are diverse. In the case of ibuprofen, better particle size results were achieved employing the H 69 process. These results were 170 nm with the H 69 process and 636 nm with the H 42 technology. However, the homogenizing equipment was different: the Avestin C5 for 10 cycles at 1200 bar in the case of the H 69 process and the Micron LAB 40 for 20 cycles at 1500 bar in the case of the H 42 process [38, 39]. Ibuprofen was also processed employing a microprecipitation-sonication technique. However, this approach led to bigger mean particle sizes than with the other combinative approaches (702 nm) [4].

With HCA as a model drug, the best results were achieved with the H 42 process with a final mean particle size of 281 nm after 20 cycles at 1500 bar. However, the H 96 process achieved a final mean particle size of 414 nm after 10 cycles at 1500 bar. Finally, the H 69 process produced with HCA a nanosuspension with a mean particle size of 787 nm after 20 cycles at 1500 bar. The latter result was considerably bigger than with the first two techniques [2, 37].

In the case of resveratrol as a model compound, the H 69 combinative processes led to a final mean particle size of 150 nm and the H 42 process achieved a final mean particle size of 200 nm. However, the homogenization conditions were different: the H 69 process was performed with an Avestin C5 for 10 cycles at 1200 bar and the H 42 technology was performed with a Micron LAB 40 for 1 cycle at 1500 bar [38, 40].

With meloxicam as model drug, a “Nanoedge-like” approach employing either sonication or HPH as the annealing step led to similar mean particle sizes (259 nm and 212 nm, resp.) [32].

In general, the particle size reduction effectiveness depends on several factors: the technology and equipment employed, as well as the physicochemical characteristics of the drug, such as solid state behavior, hardness, porosity, and morphology. A technique that produces amorphous drugs and/or highly brittle, porous, and friable structures can also lead to smaller particle sizes after the comminution step [44].

## 6. Performance Comparison between Combinative and Standard Technologies

**Figure 2:**
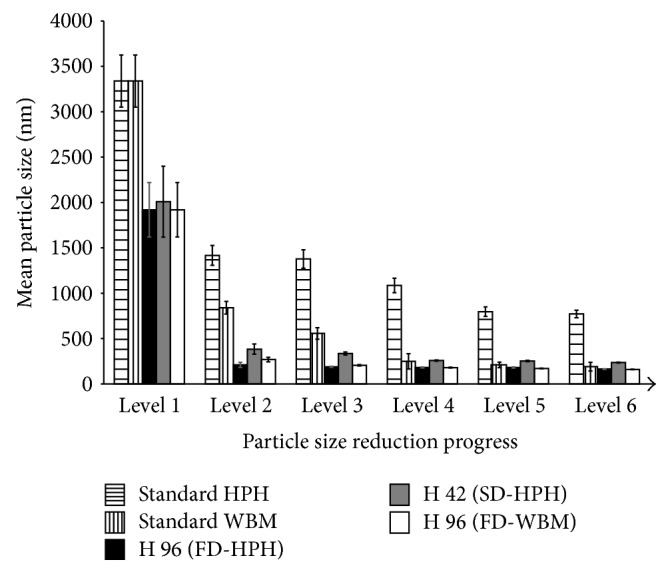
Particle size reduction performance of standard and combinative technologies. Six levels: premilling (1), 1 HPH cycle at 1500 bar/1 hour of WBM (2), 5 cycles/2 hours (3), 10 cycles/4 hours (4), 15 cycles/8 hours (5), and 20 cycles/24 hours (6).

The particle size reduction performances of standard and selected combinative processes with glibenclamide as a model compound are compared in Figure 2. The graphic description shows the superior particle size reduction effectiveness of the combinative technologies regarding the process length to achieve a nanosuspension and the smallest final mean particle size. The HPH and WBM standard techniques achieved a final mean particle size of 772 nm and 191 nm at the end of their respective processes (after 20 cycles of HPH and 24 hours of WBM). However, these processes presented a slower particle size reduction progress than the combinative methods.

When the H 96 technology (black columns) was employed, the nanosuspension had a mean particle size of about 200 nm after one cycle of HPH. At this point, the standard HPH presented a mean particle size of 1417 nm. Additionally, the standard WBM presented a mean particle size of 840 nm after one hour of milling. This mean particle size result of 200 nm after only one cycle of HPH was markedly improved than with standard HPH (772 nm) and almost the same as standard WBM (191 nm) till the end of these processes. Finally, the H 96 process achieved a final mean particle size of 164 nm after 20 HPH cycles.

When the H 96 technology was employed with WBM as the top-down step (white columns), the nanosuspension had a mean particle size of 269 nm after only one hour of milling and 160 nm after 24 hours of processing. Both results were also markedly improved compared to the standard approaches.

In the case of processing glibenclamide with the H 42 technology (grey columns), the nanosuspension had a mean particle size of 384 nm after one cycle of HPH and a final mean particle size of 236 nm after 20 cycles of homogenization. Again, these particle size results were improved compared to the standard techniques employing untreated drug material.

In general, the combinative particle size reduction processes perform faster than the standard methods to produce nanosuspensions and achieve smaller final mean particle sizes.

## 7. Conclusion

The application of nanotechnology in pharmaceutical development has great potential for the formulation of poorly water-soluble compounds. This approach has already proven to be successful with a steadily increasing number of marketed products. Universal, efficient, and easily applicable processes are the most suitable technologies for the formulation of poorly soluble drugs.

The combinative particle size reduction technologies have addressed the drawbacks of the standard techniques. The combinative processes lead, in general, to faster top-down process steps, improved physical stability, and smaller particle sizes than the standard comminution processes such as high pressure homogenization or wet bead milling. The small particle sizes have a direct impact on the dissolution rate and bioavailability of poorly soluble drugs after oral, topic, and I.V. administration. This implies improved in vivo performance. More research needs to be performed, however, to solve the technical challenges of the different technologies in order to achieve improved particle size reduction effectiveness and better formulations for new, problematic compounds. In the future, it is expected that more screenings will be performed employing the principle of design of experiments to systematically analyze the critical factors for the production of nanosuspensions. In this way, it will be possible to establish optimal process parameters to achieve final mean particle sizes below 100 nm for a wide variety of compounds.

## Figures and Tables

**Table 1:** Different drugs processed with the combinative particle size reduction technologies.

Combinative technology	Pretreatment	Particle size reduction technique	Drug	Smallest reported mean particle size	Length of the nanosuspension production	Administration focus	Reference
NANOEDGE	Microprecipitation	HPH	Paclitaxel	200 nm	20000 psi for 30 min (Avestin C50)	I.V. (reformulation as nanosuspension to eliminate cremophor EL as excipient)	[27]
NANOEDGE	Microprecipitation	HPH	Nabumetone	930 nm	20000 psi for 30 min (Avestin C50)	I.V.	[25]
NANOEDGE	Microprecipitation	HPH	Prednisolone	640 nm	10000 psi for 15 min (Avestin C50)	I.V.	[25]
NANOEDGE	Microprecipitation	HPH	Carbamazepin	400 nm	20000 psi for 15 min (Avestin C50)	I.V.	[25]
NANOEDGE	Microprecipitation	HPH	Itraconazol	581 nm	20000 psi for 30 min (Avestin C50)	I.V.	[29]
NANOEDGE	Microprecipitation	Sonication	Itraconazol	177 nm	1 min 10000 Hz (400 W)	I.V.	[25]
“Nanoedge-like”	Microprecipitation	HPH	Meloxicam	212 nm	15 cycles 500 bar (ATS AH110D)	Oral	[32]
“Nanoedge-like”	Microprecipitation	HPH	Isradipine	469 nm	30 cycles 1200 bar (GEA Niro Soavi)	Oral	[31]
“Nanoedge-like”	Microprecipitation	HPH	10-hydroxycamptothecin (10-HCPT)	131 nm	20 cycles 1000 bar (ATS AH110D)	Oral	[30]
“Nanoedge-like”	Microprecipitation	Sonication	Hydrocortisone	80 nm	5 min	Oral	[36]
“Nanoedge-like”	Microprecipitation	Sonication	Ibuprofen	702 nm	60 min	Oral	[4]
“Nanoedge-like”	Microprecipitation	Sonication	Nitrendipine	209 nm	15 min 20000 Hz (400 W)	Oral	[33]
“Nanoedge-like”	Microprecipitation	Sonication	All-trans retinoic acid	155 nm	30 min	Oral	[34]
“Nanoedge-like”	Microprecipitation	Sonication	Meloxicam	259 nm	20 min (300 W)	Oral	[32]

H 69	Cavi-precipitation	HPH	Ibuprofen	170 nm	10 cycles 1200 bar (Avestin C5)	Oral	[38]
H 69	Cavi-precipitation	HPH	Hydrocortisone acetate (HCA)	787 nm	20 cycles 1500 bar (Micron LAB 40)	Oral	[37]
H 69	Cavi-precipitation	HPH	Resveratrol	150 nm	10 cycles 1200 bar (Avestin C5)	Oral	[38]
H 69	Cavi-precipitation	HPH	Omeprazol	921 nm	20 cycles 1500 bar (Micron LAB 40)	Oral	[37]
H 69	Cavi-precipitation	HPH	Prednisolone	22 nm	1500 bar for 6 min (Micron LAB 40)	Oral	[37]

H 42	Spray-drying	HPH	Amphotericin B	172 nm	20 cycles 1500 bar (Micron LAB 40)	Oral	[39]
H 42	Spray-drying	HPH	Glibenclamide	236 nm	20 cycles 1500 bar (Micron LAB 40)	Oral	[3]
H 42	Spray-drying	HPH	Hydrocortisone acetate (HCA)	281 nm	20 cycles 1500 bar (Micron LAB 40)	Oral	[2]
H 42	Spray-drying	HPH	Ibuprofen	636 nm	20 cycles 1500 bar (Micron LAB 40)	Oral	[39]
H 42	Spray-drying	HPH	Resveratrol	200 nm	1 cycle 1500 bar (Micron LAB 40)	Oral	[40]

H 96	Freeze-drying	HPH	Amphotericin B	62 nm	5 cycles 1500 bar (Micron LAB 40)	Oral	[41]
H 96	Freeze-drying	HPH	Glibenclamide	164 nm	20 cycles 1500 bar (Micron LAB 40)	Oral	[44]
H 96	Freeze-drying	HPH	Cyclosporin A	440 nm	15 cycles 1500 bar (Micron LAB 40)	Oral	[41]
H 96	Freeze-drying	HPH	Hydrocortisone acetate (HCA)	414 nm	10 cycles 1500 bar (Micron LAB 40)	Oral	[41]

CT	Pearl milling	HPH	Rutin	604 nm	1 cycle 100 bar (Avestin C50)	Topical/oral	[6]
CT	Pearl milling	HPH	Hesperidin	599 nm	5 cycles 1000 bar (Micron LAB 40)	Topical/oral	[6]
CT	Pearl milling	HPH	Apigenin	275 nm	1 cycle 300 bar (Avestin C50)	Topical/oral	[6]
